# Human Retrotransposons and Effective Computational Detection Methods for Next-Generation Sequencing Data

**DOI:** 10.3390/life12101583

**Published:** 2022-10-12

**Authors:** Haeun Lee, Jun Won Min, Seyoung Mun, Kyudong Han

**Affiliations:** 1 Department of Bioconvergence Engineering, Dankook University, Yongin 16890, Korea; 2 Department of Surgery, Dankook University College of Medicine, Cheonan 31116, Korea; 3 Department of Microbiology, College of Science & Technology, Dankook University, Cheonan 31116, Korea; 4 Center for Bio Medical Engineering Core Facility, Dankook University, Cheonan 31116, Korea; 5 HuNbiome Co., Ltd., R&D Center, Seoul 08507, Korea

**Keywords:** transposable elements, retrotransposons, next-generation sequencing (NGS), computational tools

## Abstract

Transposable elements (TEs) are classified into two classes according to their mobilization mechanism. Compared to DNA transposons that move by the “cut and paste” mechanism, retrotransposons mobilize via the “copy and paste” method. They have been an essential research topic because some of the active elements, such as Long interspersed element 1 (LINE-1), *Alu*, and SVA elements, have contributed to the genetic diversity of primates beyond humans. In addition, they can cause genetic disorders by altering gene expression and generating structural variations (SVs). The development and rapid technological advances in next-generation sequencing (NGS) have led to new perspectives on detecting retrotransposon-mediated SVs, especially insertions. Moreover, various computational methods have been developed based on NGS data to precisely detect the insertions and deletions in the human genome. Therefore, this review discusses details about the recently studied and utilized NGS technologies and the effective computational approaches for discovering retrotransposons through it. The final part covers a diverse range of computational methods for detecting retrotransposon insertions with human NGS data. This review will give researchers insights into understanding the TEs and how to investigate them and find connections with research interests.

## 1. Introduction

In the 1950s, discrete DNA pieces, which are called “Transposable elements (TEs)”, that move within genomes, were discovered by Barbara McClintock [1,2]. Compared to protein-coding genes that make up only 1.5% of the human genome, TEs consist of nearly 45% of the entire human genome [3,4]. Since the actual function of TEs was obscured, people called TEs “dark matter,” and deciphering their roles in humans have become crucial for understanding genome evolution, genetic diversity, gene regulation, and diseases [3,5,6,7,8].

**Figure 1:**
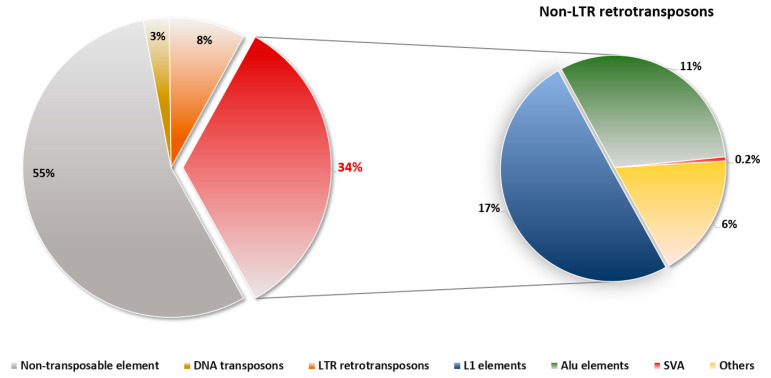
Composition of non-LTR retrotransposons in the human. Non-LTR retrotransposons constitute approximately 34% of the entire human genome. A total of 17% of L1 elements, 11% of *Alu* elements, and 0.2% of SVAs belong to non-LTR retrotransposons. As some of the elements are still active in humans, they cause genetic disorders and contribute to diversity.

TEs can be classified into two classes depending on their transposition concept: DNA transposons and retrotransposons [9,10]. DNA transposons move by the “cut and paste” mechanism in the human genome, involving excision and reinsertion at different sites [9,11]. They account for 3% of the human genome, but there is little information on a human because they are currently inactive [1]. In contrast, retrotransposons have been the critical subject of extensive studies until today because active retrotransposons diversify human genomes by regulating gene expression and novel retrotransposon-mediated mutations [12]. Retrotransposons generally mobilize via the “copy and paste” mechanism, which involves the transcription of an RNA intermediate and insertion into new sites with the form of a cDNA copy [11,13]. Retrotransposons are categorized by whether they contain long terminal repeat (LTR) structures, and their flanking regions have regulatory sites such as promoters, polyadenylation, and enhancers [11,14,15]. Typically, the LTR elements in humans are human endogenous retroviruses (HERVs) which account for 8% of the human genome [1,16]. In contrast, non-LTR elements such as Long interspersed element 1 (LINE-1), *Alu*, and SVA elements, which comprise one-third of the human genome, have shown activities and cause genomic diversity, genetic alteration, and associated diseases [17,18,19] (Figure 1).

In line with previous studies that reported SVs, especially insertions, in human genomes related to genetic disorders [20,21,22,23,24], various bioinformatics tools to detect retrotransposon insertions have been developed over a few decades [25,26,27,28,29,30]. Furthermore, when considering next-generation sequencing (NGS) technology, which has increased the possibility of genome research, the investigation of retrotransposons is also accelerating [31,32]. Therefore, this paper addresses the non-LTR retrotransposons, representative NGS platforms, and diverse computational methods to detect retrotransposons in humans. This review article will give researchers extensive insights into how retrotransposons have affected the human genome and which bioinformatics tools should be utilized to detect retrotransposon insertions, depending on the research purpose.

## 2. Non-LTR Retrotransposons in Humans

### 2.1. LINE-1 (L1) Elements

**Figure 2:**
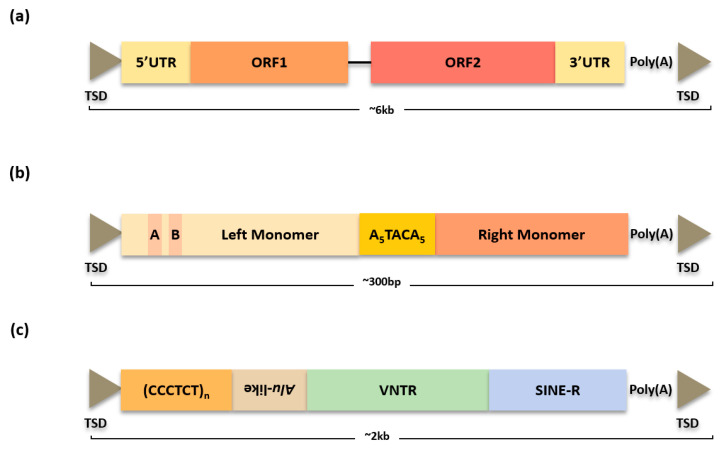
Structure of L1, *Alu*, and SVA elements. (**a**) L1 elements have ~6 kb length. ORF1 in L1 element encodes RNA-binding protein. ORF2 encodes endonuclease and reverse transcriptase for self-mobilization. (**b**) *Alu* elements have a dimeric structure with a length of 300 bp. The two monomers are present on both sides of the A-rich region. (**c**) The canonical length of SVA elements is 2 kb. They contain five distinctive regions.

L1 elements contribute approximately 17% of the entire human genome and have ~6 kb long length [1]. L1 elements consist of a 5′ untranslated region (UTR) with an internal RNA polymerase II, two open reading frames (ORF1 and ORF2), 3′UTR, and end with a poly (A) tail [33] (Figure 2a). The ORF1 and ORF2 play different roles in L1 activity. The ORF2, which encodes endonuclease and reverse transcriptase, serves as L1 machinery functions for self-mobilization. On the other hand, ORF1, characterized by three distinct domains, encodes RNA-binding protein. Even though the precise function is not fully understood, a study suggests that the strongly conserved long coiled-coil (CC) domain of the N-terminal domain contains cysteine residue layers that enable ORF1 to bind to various metal ions [34,35,36]. Both ORF1 and ORF2 proteins generally show *cis* preference, producing a ribonucleoprotein (RNP) particle that enables L1 to maintain its ability despite numerous non-functional elements [37]. There are more than 500,000 copies in the human genome that were propagated ~150 million years ago [38]. During evolution periods, most of the L1s became inactive due to the accumulation of mutations because the majority of L1 insertions are pseudogenes [39,40]. However, approximately 80–100 human-specific L1s (L1Hs) are still competent, and each individual’s L1 elements have not been fully recorded in the reference, which still contribute to genetic diversity [13,33,39,41].

L1s can mobilize the human genome via a target-primed reverse transcription (TPRT) mechanism. The process begins with the cleavage of the first strand at 5′-TTTTAA-3′ sites by the L1 endonuclease. The 3′ exposed hydroxyl (OH) is then utilized as a primer for L1 reverse transcription. The second strand of the target site is nicked and finally synthesized. This process involves frequent 5′ truncations and a 3′ end poly (A) structure. Newly inserted L1 elements have hallmark features of 2~20 base pairs flanking target site duplications (TSDs) [1,42]. The analysis of the initial human genome reported 41% of GC contents, but L1 insertion regions showed approximately 36–38% in some studies [43,44,45]. Thus, most researchers estimated that L1 endonuclease prefers to cleavage AT-rich regions. However, in 2019, Shin et al. confirmed that non-reference L1Hs insertion sites showed 41.15% of GC contents, implicating that L1 elements are randomly integrated into an Individual genome [39,44].

In 1988, there was the first report on a novel L1 insertion in a patient by Haig Kazazian and his colleagues. Although the patient with hemophilia A had no familial history, exonic L1 insertion in X-linked gene factor VIII indicated the cell types that can be inherited by the next generation [18,24]. Since more than 100 reports have addressed heritable diseases related to L1 insertions, L1s were regarded as inactive in adult somatic tissues [1,18,46]. However, retrotransposition is not limited to the germline. Recent research has determined that L1s can initiate retrotransposition which results in somatic arrangements during neural development and in epithelial cancers, concluding that there are more widespread retrotranspositions than predicted [18,46,47,48].

### 2.2. Alu Elements

*Alu* elements are one of the most prevalent and numerous TEs in the primate genomes, showing more than 1.1 million interspersed copies in humans [6]. The human genome has an average of one *Alu* copy per 3 kb, making it the most successful TE in terms of copy number [1]. The origin of *Alu* elements is the 7SL RNA gene, which is responsible for protein secretion as a component of the signal recognition particle (SRP) [49]. The canonical length of the *Alu* element is ~300 bp, which has a dimeric structure, and two separate monomers are connected by an A-rich region tract [50] (Figure 2b). This dimer structure of *Alu* evolved through a multistage procedure that formed and united its ancestral monomer. It is reported that ribonucleoprotein (RNP) modulates RNA stability [51]. Several intact dimeric *Alu* RNA molecules give rise to stable left monomer transcripts, named small cytoplasmic *Alu* (sc*Alu*) [51,52]. In vivo, *Alu* and sc*Alu* transcripts interact with the *Alu*–RNA binding subunit and stabilize sc*Alu* RNP by binding SRP9 and SRP14 proteins [53,54]. In contrast to the left *Alu* monomer maintaining an affinity for SRP9 and SRP14 proteins, the right monomer is relatively less stable and shows a loss of affinity for SRP9/14. Given that affinity for the SRP9/14 protein is related to sc*Alu* RNA production in vivo, the stability of the right monomer is an important parameter that can determine the competency of the *Alu* element during its genome evolution [51,53,55].

The 5′ region located in its left monomer contains an RNA polymerase III promoter and ends with a 3′ region followed by an A-rich tail [1,6]. The *Alu* itself does not contain the RNA polymerase III terminator signals, and thereby transcription extends to the downstream 3′ flanking sequence near the TTTT location [56]. *Alu* has sometimes been criticized as being a ‘genomic parasite’ because *Alu* elements are non-autonomous, which means they do not have a coding capacity [57,58]. Therefore, they utilize the retrotransposition enzymatic capacity of L1s. However, where *Alu* RNPs access the L1 machinery (whether the cytoplasm or nucleus) is still uncovered. Nevertheless, *Alu* RNPs are primarily found in the cytoplasm and make use of L1 proteins in the *trans* form to mobilize in the genome [59].

*Alu* elements are classified into three major subfamilies: *Alu*J, *Alu*S, and *Alu*Y, based on diagnostic mutations over millions of years [60]. The *Alu*J lineage is the oldest, followed by the *Alu*S family and the *Alu*Y family. Since these subfamilies have different genetic ages, it can be inferred that each subfamily has integrated into the human genome at other times [61]. In addition, the primate lineages where specific subfamilies have shown activities indicate particular insertion patterns. The subfamilies can be shared between related taxonomies, but several studies have verified species-specific elements [62,63,64]. In 2015, Konkel et al. described the details of 332 unique *Alu* variants in humans because active *Alu* elements such as *Alu*Ya5 and *Alu*Yb8 still contribute to SVs, especially by insertion [65]. The insertions are found in exons, introns, and 3′ UTRs and cause genetic diseases such as Hyper IgM (HIGM) Syndrome, leukemia, and breast cancer [66,67,68]. As novel *Alu* insertions continue to generate 0.1% of human genetic diseases, *Alu* amplification will contribute to population genetic diversity and disorders [67,69].

### 2.3. SINE-VNTR-Alu (SVA) Elements

SVA elements were derived ~25 million years ago in hominids, representing the youngest retrotransposon in the human genome [1,70]. They have a 2 kb length and are composed of a (CCCTCT)n hexamer simple repeat, an *Alu*-like region, a variable number of tandem repeats (VNTR), a short interspersed element of retroviral origin (SINE-R), and oligo(dA)-rich regions [71] (Figure 2c). There are ~3000 elements in the human genome, and they are highly enriched in the G + C-rich regions [72]. The L1 trans proteins also mobilize SVA elements because they are non-autonomous TEs like *Alu* elements [73].

Since the development of bioinformatics and sequence analysis, there have been advancements in our understanding of SVAs [74]. Additionally, as SVAs are currently active in humans along with L1s and *Alu* elements, they are occasionally inserted into genes and cause genetic diseases such as Hemophilia B and X-linked Dystonia Parkinsonism [58,75,76]. Consequently, even if the SVA components account for only ~0.2% of the entire genome [76,77], SVA insertion detection and elucidation are continuously required.

## 3. Representative Next-Generation Sequencing (NGS) Platforms

In recent years, sequencing technology evolution, such as NGS, has improved the potential of genomic studies [78,79,80]. In particular, the discovery of genes associated with human disease and the genetic variants associated with them has accelerated, as NGS provides rapid, sensitive, accurate, and cost-effective genetic testing [81,82,83,84]. Since the Human Genome Project (HGP), several genome projects such as the 1000 Human Genomes Project (http://www.1000genomes.org/ (accessed on 14 May 2022)), the International HapMap Project, and the Cancer Genome Anatomy Project (http://cgap.nci.nih.gov/ (accessed on 14 May 2022)) have accumulated a vast amount of sequence data about the human genome and enabled scholars to gather information about genetic variations such as insertions and deletions (INDELs), single-nucleotide polymorphisms (SNPs), copy number variations (CNVs), and SVs mediated by retrotransposons [85,86,87,88,89]. Taken together, we will introduce representative and prevalent NGS platforms currently utilized for genomic research, including retrotransposons.

### 3.1. Illumina

The first short-read sequencing platform was introduced in 2006 by the Solexa Genome Analyzer, which was incorporated by Illumina later [90]. The basic principle of the Illumina platform is “bridge amplification”, in which about 500 bp DNA molecules with specific adapters at both ends are arched and repeatedly amplified on a glass slide [91,92]. The iterative amplification procedure generates “clusters” made up of 1000 copies of each fragment on the glass slides, and each glass slide includes numerous spaced oligonucleotides complementary to the adapter sequence, thus supporting millions of parallel reactions [92]. During the sequencing, respective modified nucleotides with a unique fluorescent label are induced into the synthesis reactions and are subsequently detected.

The variety of applications of Illumina support research areas such as cancer, microbiology, agricultural genetics, and molecular biology, as well as clinical areas such as reproductive health, rare diseases, and oncology. Versatile instruments (iSeq 100, MiniSeq, MiSeq, NextSeq 550, and NovaSeq) have been launched to meet different purposes of studies [93]. As each sequencer provides a different range of uses (including transcriptomics, genomics, and epigenomics), it is important to adopt appropriate instruments for optimal results. More detailed information about equipment capabilities can be found in (Table 1). In 2014, McCoy et al. tested Illumina’s TruSeq synthetic long-reads technology that can achieve lengths of 1.5~18.5 kb with a low error rate (~0.03% per base). Since TruSeq correctly identified 77.8% of the annotated TEs of Drosophila melanogaster, it is anticipated to be a practical approach for understanding the dynamics of TEs, a ubiquitous feature of many species, including humans [32].

The shortage of synchronization in the synthesis reactions among the various clusters is a potential problem with the Illumina sequencer, which can produce an inaccurate consensus sequence. Thus, the amount of template DNA must be precisely quantified to avoid “overclustering” [92]. Notwithstanding, the Illumina platforms are the most widespread methods owing to their high accuracy (>99%), relatively low cost, and high throughput [94]. Overall information about Illumina is available at (https://www.illumina.com/ (accessed on 10 July 2022)).

### 3.2. MGI

An affiliated group of the Beijing Genomics Institute (BGI), MGI Technology, released a series of NGS machines (MGISEQ-2000, DNBSEQ-T7, DNBSEQ-G400, DNBSEQ G50) (Table 1) [95]. MGI’s sequencing technology includes a peculiar single-strand circular library construction method. During the sequencing library preparation, double-stranded DNA with adapters at the ends is heated and generates single-strand DNA. After a complementary sequence to both of the 5′ and 3′ ends is hybridized to single strand DNA, DNA ligase repairs a nick and forms DNA nanoballs (DNBs) via rolling circle amplification (RCA) with the Phi 29 DNA polymerase using the single strand as a template. In the sequencing step, each DNB is loaded into a distinct section of the nanoarray patterned flow cell with a positive charge, allowing only one DNB per active site [96]. Finally, the fluorescent signal is converted to digital information based on a combinational probe anchor synthesis (cPAS) sequencing [97]. The advantages of low amplification error rates from the DNBs library and high density patterned nanoarray technology dramatically improve sequencing accuracy and reduce duplication rate.

MGI platforms support reproductive health, whole genome sequencing, whole exome sequencing, microbial detection, tumor, plant, RNA, and forensic genomic areas according to (accessed 12 July 2022, https://en.mgi-tech.com/). Several studies investigated the sequencing quality of MGI platforms compared to Illumina equipment to gauge their compatibility with the Illumina one. For example, in 2021, Jeon et al. explored the whole genome sequencing of normal Korean tissues and those with lung tumors using Illumina NovaSeq600, MGISEQ-2000, and DNBSEQ-T7. After sequencing, they compared and evaluated the sequencing performance for variant calling, including single nucleotide variants (SNVs), insertions, and deletions, and confirmed that the DNBSEQ-T7 could detect a few more significant number of indels than NovaSeq 6000 [98]. Furthermore, Lang et al. validated that MGISEQ-2000 found 101~133 bp loss, which was missed by NextSeq500 [99]. These recent research results suggest that MGI platforms are highly concordant with the Illumina instruments.

In 2019, second-generation sequencing-based single-tube long fragment read (stLFR) technology capable of sequencing from long DNA molecules (10~350 kb) was described. The stLFR technology exploits transposome-containing Tn5 transposase and two different purposes of transposons (a single-stranded one for hybridization with bead capture splint oligo and a double-stranded one for recognizing enzyme and transposition reactions). Using transposome enables the insertion of a hybridization sequence approximately every 200–1000 bp on long genomic DNA. Next, the TE-integrated DNA molecules are hybridized to a bead that contains ~400,000 copies of an adapter containing a unique barcode, PCR primer site, and capture sequence complementary to the integrated transposons. After the library construction, these co-barcoded subfragments are examined using MGISEQ-2000 or equivalent [100]. Since the stLFR is based on adding identical barcode sequences to subfragments of long DNA molecules (DNA co-barcoding), it might be a tentative efficient method to detect TEs in humans [100].

### 3.3. PacBio and Nanopore

While the second-generation sequencers have improved significantly in sequencing data yield and production speed compared to Sanger sequencing, they have some limitations, especially their short read-length capability. Thus, they are still not adapted for understanding biological challenges, such as gene isoform, methylation, and complex genomic regions [101]. Hence, in the early 2010s, a novel set of third-generation sequencing methods were introduced: Pacific BioSciences (PacBio) and Oxford Nanopore Technologies (ONT), which currently dominate the long-read sequencing areas [102,103]. Contrary to the prior sequencing technologies that rely on PCR methods to amplify a given template, the third-generation sequencers have two distinctive features that can ameliorate biases resulting from the PCR procedure. First, they allow for analysis in real time, and second, they interrogate a single molecule of DNA with no need for synchronization [102,104,105]. As the read length of the third-generation sequencers is much longer than that of second-generation sequencing technologies with maximal lengths of 30–150 kb, it is expected to be established as a more applicable method to detect various SVs, especially derived from retrotransposons [106,107,108,109].

The PacBio sequencer, also known as the single molecule real-time (SMRT) sequencing method, exploits template-oriented synthesis using four differently fluorescently labeled deoxyribonucleoside triphosphates (dNTPs) [110]. For the conduction, a circular “SMRTbell” adapter is necessary [102]. Compared to other second-generation NGS technologies where polymerase travels along a template, SMRT sequencing utilizes a microscopic chamber named zero-mode waveguides (ZMWs) that immobilize DNA polymerase with a single strand template [102,111]. The ZMW chamber, including a sensor and a camera, then records the signal generated by integrating phosphate-labeled dNTPs at the elongated strand. The detection is identified when each base binds to the growing chain, timed to coincide with the incorporation of the nucleotides [92]. As summarized in Table 2, the PacBio technology can generate a 10 Gb output for the RSII platform and a 500 Gb output for Sequel systems [112]. The information is available at (https://www.pacb.com/ (accessed on 15 July 2022)).

Nanopore sequencing technology relies on nanoscale ‘nanopore’ proteins acting as biosensors encased in an electrically resistant membrane (https://nanoporetech.com/ (accessed on 3 July 2022)). [113]. When double-stranded DNA molecules are denatured, a single-stranded DNA or RNA molecule passes through a nanopore electrode, and then the changes in electronic current are detected and measured [114]. After the detection, a basecalling process of converting the ion current into sequences is performed. However, nanopore basecalling systems do not directly sequence each individual base. Instead, the sequencing is conducted 5-mers, indicating that up to ~1024 incorrect signals can be formed [115]. Even though concerns about accuracy still exist because the platform has an average error rate of 5% to 13%, the nanopore technology is useful for constructing a genome backbone of unknown organisms and supports a wide range of applications, such as pathogen detection in plant viruses and SV detection in cancer (Table 2) [113,114]. Furthermore, nanopore technology allows for unmodified DNA, and thus the processing speed is fast (<3 h) [104,116].

## 4. Computational Methods to Detect Retrotransposons in Humans Based on NGS

### 4.1. Short-Read Sequencing Data

#### 4.1.1. RetroSeq

**Figure 3:**
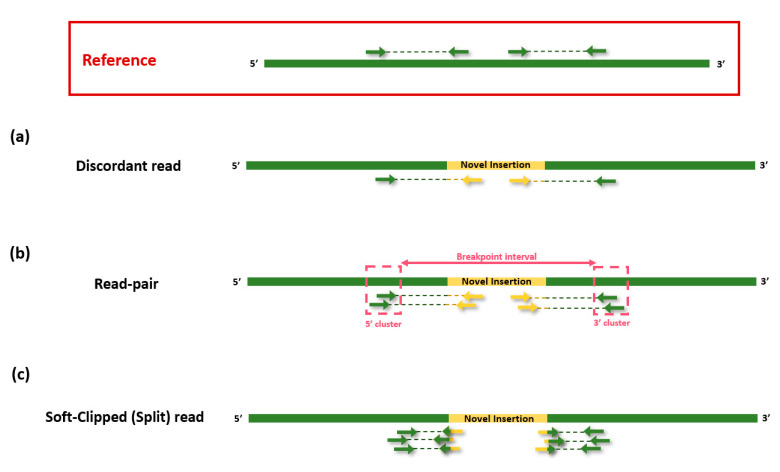
Illustration of discordant read, read-pair and soft-clipped reads. (**a**) A discordant read indicates that one-end read is fully mapped to the reference, but another end is not mapped to the reference. (**b**) A read pair provides putative insertion site and gives information about breakpoint interval based on 5′cluster at one end and 3′cluster at the other end. (**c**) A soft-clipped read (split read) refers a mate pair where one part is partially mapped to the reference. Hence, the truncated read contains both reference and novel insertion sequences.

In the early 2010s, a wide range of bioinformatics tools such as VariationHunter and Hydra were developed to find non-reference TE insertions [117,118]. Following these technologies, Keane et al. introduced new software, RetroSeq, which is used to detect non-reference TE insertions from Illumina paired-end whole-genome sequencing (WGS) data [119]. RetroSeq has two phases. The first is the discovery phase, in which discordant mate pairs are matched and categorized into TE classes (L1, *Alu*, SVA, etc.) by either using the reference’s annotated TEs or aligning with the exonerate program (Figure 3a) [120]. Then, in the second, the calling phase uses the anchoring mates of the TE candidates read in the previous step and clusters them based on their genomic location and aligned strands. When the forward or reverse strand clusters created from the anchor reads merge into presumed breakpoints, RetroSeq employs any available soft-clipped reads to further refine the TE insertions’ breakpoints by profiling the density of the matching forward and reverse clusters. Based on the trio samples of central European (CEU) used in the subsequent study of the 1000 Genomes Project (NA12891, NA12892, and NA12878), RetroSeq was found to have an average sensitivity of 97% and 83% for detecting *Alu* and L1 elements, respectively [70,119]. In addition, Helman et al. demonstrated somatic retrotransposon insertion in exonic, intronic, and intergenic regions, showing 99% specificity with cancer WGS data [121]. In summary, RetroSeq can be utilized to investigate novel TE insertions with WGS data, provided an appropriate reference genome is available.

#### 4.1.2. Alu-Detect

In 2013, the alu-detect tool was developed to find novel *Alu* elements and their precise breakpoints by David et al. from WGS and whole-exome sequencing (WES) data. The major steps of the alu-detect are as follows: First, the read fragments that are poorly mapped, reads tails, and discordant pairs are selected by mapping to the reference genome. These fragments (reads or read pairs) are collected and then remapped to the reference genome so that the location of the insertion breakpoints can be detected. Second, they are reused to determine evidence of *Alu* insertions by mapping to the set of consensus *Alu* sequences (available at dbRIP), followed by a phase in which the clusters of the read fragments along with evidence of *Alu* insertions are constructed [122]. In that phase, the orientation and relative position of the paired end are also considered for precise detection. Finally, a split mapping algorithm that enables alignment to leap from the reference to an *Alu* sequence and back is performed for each read in the clusters. When the non-reference *Alu* insertion is detected with breakpoints, they are nominated as a novel *Alu* insertion based on the thresholds such as the mapping quality, length of the *Alu* insertion, and the number of reads [123].

In order to evaluate the accuracy and the recall rates, seven people were selected as follows: a trio of Yoruban (NA18506, NA18507, and NA18508) and CEU (NA12891, NA12892, and NA12878) and an unreported ancestor (SRS228129). According to Illumina 100 bp paired-end WGS data from seven people, there were on average 1718 and 1339 *Alu* calls per Yoruban and CEU, respectively. Furthermore, it shows approximately 85% recall and 97% accuracy, respectively, identifying 1519 novel *Alu* insertions on average that were previously not reported in the reference. The difference between alu-detect and other TE detection software tools is that it is only focused on *Alu* elements that are still active, and TSD detection at the breakpoint of *Alu* insertion and tool running using both WGRS and WES data are possible [124]. Furthermore, the alu-detect is sensitive enough to identify *Alu* insertions adjacent to other *Alu* elements. 

#### 4.1.3. Tangram

Although RetroSeq exhibits high sensitivity and specificity in retrotransposon detection, there is a limitation in analyzing only split reads, even when the read pairs suggest a potential insertion site [125]. Tangram, which contributes to detecting TEs in the 1000 Genomes Project, is an effective program that integrates soft-clipped (split) reads and read pairs. One of the representative characteristics of Tangram is the ability to pinpoint the breakpoints with single-nucleotide accuracy. In addition, it can simultaneously process the insertion detection steps of various fragment lengths at the population scale. There are two methods to find the breakpoint based on the type of reads. In the case of the read pair, uniquely mapped reads at 5′ clusters and the other aligned mate pairs at the 3′ clusters are used to estimate the breakpoint interval (Figure 3b). For the soft-clipped read, the reads that one mate pair is aligned but partial of another mate pair is unaligned or feature of soft-clipped are investigated (Figure 3c). As these reads are split into two parts (segments mapped onto the human reference and the consensus *Alu* sequence), the first segment is used to identify the breakpoint location [126]. In terms of Tangram, the split-read mapping stage is performed prior to the read-pair mapping step so that they can “nucleate” the SVs at the outset [125].

To evaluate the ability of Tangram, Wu et al. analyzed the precision of the *Alu* and L1 detection and genotype calling using WGS data of CEU (NA12891, NA12892, and NA12878) samples that have an average of 81X coverage. As a result, Tangram discovered *Alu* elements with more than 97% sensitivity. In addition, it showed more than 91% accuracy in genotype, enabling a distinction between heterozygotes and homozygotes. Regarding L1s, it showed more than 91% sensitivity and 91% genotype accuracy. These figures for detection accuracy are relatively higher compared to RetroSeq, which indicated 78% sensitivity and 66% genotype accuracy. Furthermore, 23 samples were utilized for a specificity investigation including the CEU trio and low-coverage (~5X) populations. Tangram detected 2874 *Alu*, 256 L1, 53 SVA, and 22 HERV-K insertions, of which 357 insertions were novel. Since Tangram had a low false positive rate under 6%, it is expected to be used to uncover new insertions with a varied range of clinical samples [125].

#### 4.1.4. Mobile Element Locator Tool (MELT)

As mentioned above, L1, *Alu*, and SVA elements can move within the human genome by L1′s retrotransposition mechanism, TPRT. Consequently, they have a common component called TSDs, which has been used as a hallmark feature in detecting retrotransposons [73,127]. In addition to the TSDs, several other uncanonical features can be utilized to detect insertions. These include 3′ transduction results from a weak 3′ polyadenylation signal, 5′ inversion caused by twin priming, and 5′ truncation by an incomplete replication L1 RNA copy [128,129,130,131].

MELT software tools aim to detect TE insertions on a population scale to construct comprehensive data worldwide and to also discover the uncanonical features which are meaningful for studying the genetic and biological effect of TE insertion. In addition, it performs genotyping for both non-reference and reference TEs. Furthermore, this tool supports the possible effects of each inserted ME on the surrounding genes and annotates the features of the affected gene (e.g., exon, intron, UTR, promoter, and terminator). First, MELT scans differentiated read pairs to determine potential non-reference insertion sites, followed by the step in which MELT utilizes split reads to nominate breakpoints and TSDs. MELT runs with WGS data and provides flexibility depending on the experimental purpose. Through modes suitable for the number of samples, they developed and provided flexibility in data implementation. The single sample (MELT-single) is suitable for a small number of samples. On the other hand, the multiple sample (MELT-split) and the multiple-sample automated (MELT-SGE) modes are appropriate for studies involving hundreds or thousands of samples [132].

MELT execution time per sample is shorter than that of RetroSeq and Tangram, showing 10.7 min per sample at NA12878 6X coverage WGS data (100 bp paired end) and 93.3 min per sample at 30X coverage WGS data (250 bp paired end). Using the 2504 low-coverage (6X–17X) genome data of the 1000 Genomes Project, MELT performed a detection of TE insertion up to 3.8 faster than Tangram at 21.9 days. Moreover, the sensitivity and specificity of the distinction were evaluated by randomly distributing 1114 retroelements (*Alu*, L1, and SVA) from the NA12878 sample. Compared to other tools, MELT showed about 100% sensitivity at 30X and 60X coverage data. Further, MELT had nearly 0% false negative rates compared to RetroSeq, which showed 60% specificity at 30X and less than 40% at 60X in detecting L1s [132].

Additionally, 121 L1 insertions derived from 3′ transductions were newly found in 2504 low-coverage human genome data sequenced by the 1000 Genome Project (GP), and 1634 samples in the 1000 GP were validated to contain 298 non-reference TE insertions with 5′ inversion. Interestingly, MELT provides flexibility and applicability to other species, such as chimpanzees and prehistoric hominids, under the condition that input data in which sequencing-completed genome data and the nucleotide sequence reference of species-specific TE insertions exist. For example, the performance of a MELT analysis with the other genomes was evaluated by detecting 7278 *Alu* and 4381 L1 insertions in 25 chimpanzees and 41 ancient *Alu* insertions in Neanderthals. Overall, MELT provides a wide-scale availability, a broad range of TE insertion features, and patterns of TE insertion inheritance with high sensitivity and specificity [132].

#### 4.1.5. IMGEins

Typically, TE detection tools using paired-end sequencing data rely on only the two approaches (discordant read pair and split-read mapping) to infer the direct breakpoints where their fragments are positioned. In addition to these features, iMGEins achieved the process of the de novo assembly of the contigs to find de novo TE insertions that are found differently for each individual. Based on the mapping status, the reads are classified into three groups: mapped reads (M), in which a one-end read is fully mapped; unmapped-reads (U), where a one-end read is not mapped to the reference; and soft-clipped reads (S), which contain both partially mapped and truncated sequences. After investigating the integrity (e.g., presence of short indels, read depth of breakpoints’ both sides, and accuracy of soft-clipped reads for breakpoints reference) of the candidate breakpoints with soft-clipped pairs, novel TE insertions are finally verified with one-end unmapped reads (i.e., M-U or U-M paired end) in the subsequent identification and assembly stage [133]. In the assembly stage, all one-end unmapped reads near the breakpoint and soft-clipped reads carrying the breakpoint are assembled using SOAPdenovo2 with the k-mer size of 51 [134]. After applying a few more algorithmic parameters (contig length, number of assembled reads in contig assembly, and mapping reads to contig), they successfully report the breakpoints, TE insertion, and valuable features of assembling and identifying the novel insertions.

In order to compare the performance of iMGEins with other cutting-edge computational methods, Bae et al. set two simulated human genomes. In the first simulation, 200 TE insertions without SNVs, 300 TEs with 10–50% SNVs, and 500 random control sequences similar in length to TE insertions were used to measure recall rates and precision. The average recall rate of the 200 TEs without SNVs and 300 TEs with SNVs from iMGEins was 97% and MELT was 98.5%, but RetroSeq had 29.3%. For the random control sequences, iMEGins had 98.6% of recall rates, while RetroSeq and MELT showed 35.1% and 0%, respectively. The second simulated human genome on chromosome 11 contained 80 known TE insertions of primates, 80 known TE insertions of humans, and 80 novel sequences. To evaluate the average precision according to different coverage, 30X and 90X WGS sequencing reads were employed. Overall, iMGEins found the most breakpoints showing 97.07% and 100% at 30X and 90X, respectively. In particular, iMGEins found novel insertions with an average of 96.8%, whereas RetroSeq and MELT could not find novel insertions at both low and high coverage. The breakpoint detection evaluation in one (NA12878) human whole genome data was also conducted. In the evaluation, iMGEins discovered 3811 breakpoints that were annotated with L1 and *Alu* elements. As a striking feature, iMGEins accurately predicted more than 90% of breakpoints within 20 bp of the annotated breakpoints. Taken together, iMGEins has important properties that can help researchers find novel or distinctive TE insertions in individuals. Therefore, it will reveal information about TE insertions relevant to population dynamics [133].

### 4.2. Alignment-Free Raw Reads

#### AluMine

All the methods described above are based on mapping sequencing reads and interpreting new insertions by split-read locations of a single read and/or the interval between paired-end reads [119,123,125,132,133]. An alignment-free computational method called AluMine can rapidly detect novel *Alu* insertions from the human WGS. Additionally, it directly genotypes from raw sequencing reads using small k-mer frequencies which contain enough base sequences from the genome and nucleotides of the *Alu* element. There are two key steps to detect polymorphic *Alu* insertions. The detection of new insertion discovery (REF-) occurred in the tested genomes but not in the reference genome and occurred in the missed *Alu* elements in the current reference genomes (REF+) but not in the tested genome. Both pipelines use 10 bp very consensus sequence from the 5′ end of the *Alu* element (GGCCGGCGC). In the REF- pipeline, all *Alu* occurrence candidates containing 25 bp flanking sequencing of raw reads are recorded and marked as a novel element if the 10 bp in the raw reads differs from the reference. On the other hand, the REF+ method utilizes a 10 bp consensus sequence to detect precise locations where the preceding 5 bp TSD sequence is duplicated 270–350 bp downstream from the signature sequence. Afterward, both pipelines generate 32-mers (25 bp to 5′ region sequences and 7 bp to either reference 3′ region sequences or consensus *Alu* insertion) at breakpoints for the genotyping of *Alu*s in each individual [135].

The effectiveness of AluMine was tested with 2241 high-coverage (30X) whole genomes from the Estonian Genome Project [136]. As a result, Puurand et al. found 13,128 REF- and 15,834 REF+ *Alu* elements. Although some of the discovered *Alu* elements were unsuitable for genotypes due to short k-mers, the concordance rate between the predicted genotypes using the tool and the experimentally observed genotypes was 98.7%. Further, a sample of NA12878 was examined, detecting 63% of the reported *Alu* elements and discovering novel 458 elements (REF-). The remaining 37% may be the truncated *Alu* elements since AluMine only covers the full-length *Alu* elements. Depending on the hardware, it takes 2 h to operate the REF- pipeline, 20 min for REF+, and from 0.4 to 4 h for genotyping per person. Based on these advantages, the alignment-free method can be applied to other TEs such as L1 and SVA elements by allowing the variable length of the TE signature sequence and k-mers [135].

### 4.3. Long-Read Sequencing Data

#### PremAsking Long Reads for Mobile Element InseRtion (PALMER)

Although the short-read-based method shows high accuracy, it still has limitations in identifying large and abundant insertions in densely repetitive genomic regions, which result in an under-representation of TEs [137,138]. One of the next-generation methods to overcome the limitations of TE insertion detection using such short-read-based sequencing data is to use long-read sequencing data produced by the PacBio platform. A PremAsking Long reads for Mobile Element inseRtion (PALMER) was first developed to detect comprehensive L1Hs insertions from long-read sequencing data in the NA12878 genome. To discover germline non-reference L1Hs, PALMER first pre-masks known retrotransposons (L1s, *Alu*, and SVA elements from Repbase, which is a web-based database consisting of eukaryotic TEs) in aligned long reads [139]. After the processing, PALMER searches for “hot L1” to detect non-reference L1Hs in the remaining unmasked genome and next selects estimated reads [140]. Then, PALMER screens 50 bp 5′ upstream and 3.5 kb 3′ downstream to identify TSDs, 5′ transduction, and poly (A) sequences. The final putative insertions should contain more than 25 bp of sequences identical to “hot L1” (L1.3; GenBank: L19088). Additionally, they should include at least 20 bp poly (A) sequences and more than 6 bp of identical TSDs [137].

Based on this approach, the WGS of the 50X coverage NA12878 sample with PacBio was applied for germline new L1Hs detection. During the process, the Canu pipeline to correct the read error was performed to improve the accuracy limitation, reducing the error rate to under 4.5% [141]. Therefore, the final 203 L1Hs candidates were validated, showing about 98.19% similarity with “hot L1”. The benchmarking assay by Zhou et al. compared PALMER with MELT to investigate the extent of the missed L1Hs detection. Of the 203 candidates, MELT identified approximately 45% (113/203) of L1Hs and missed 44.3% (90/203) that could be candidates nested within “repeat in repeat” regions [137,138].

The specificity examination of PALMER was also tested with the L1PA2 subfamily members. The L1PA2 subfamily is known to have amplified before the divergence of chimpanzees and humans [142]. The 1000 Genomes Project reference features 1544 L1Hs and 4917 L1PA2, and PALMER did not identify any new L1PA2 insertions in NA12878, demonstrating that PALMER specifically identifies the bona fide non-reference L1Hs subfamily. Notably, the pre-masking process can be extended to Oxford Nanopore Technologies. Taken together, PALMER is projected to uncover veiled retrotransposons, including *Alu* or SVA elements, that have not been previously observed [137]. In the case of PALMER, since it uses the long read of the PacBio platform, it has a sufficient detection efficiency to distinguish the L1 subfamily and is effective for different types of retrotransposons.

### 4.4. Hybrid Sequencing Data

#### x-Transposable Element Analyzer (xTea)

Several bioinformatics tools have shown high sensitivity and specificity in detecting non-reference TEs. However, the prediction and detection tools we reviewed above are designed for either short-read or long-read platforms. In 2021, a new bioinformatics tool named the x-Transposable Element Analyzer (xTea) was developed, which can be applied to short-read, long-read, and hybrid WGS data. The exceptional characteristic of the xTea software is that it can discover a wide range of retrotransposons, including L1, *Alu*, SVA, HERV insertions, processed pseudogene, and insertion-mediated SVs. Moreover, a comparative analysis of TE insertions between the germline and somatic in cancer genomics are available. Furthermore, it can be implemented at the single population level by achieving full parallelization. For short-read sequencing data (Illumina), the xTea uses both discordant paired reads and split (clipped) reads. This approach first exploits split reads that might be located close to other SVs to improve the detection accuracy rate. In addition, this step considers the alignment pattern and the precise position of the alignment read in the matched TE consensus sequence. The alignment patterns should be identical to a single breakpoint and the estimated insert size. The xTea begins with split/clipped reads and considers mechanistic signatures—the presence of TSDs and poly (A) tails for the collection of precise insertion candidates with a high confidence. For the long-read sequencing data (PacBio/Nanopore), the xTea classifies putative insertion via a split read involving partial flanking sequences of an insertion and non-split reads that contain the entire TE insertion sequences. The xTea reconstructs the entire sequence of the inserted TE and flanking regions by performing a local assembly of the collected supporting reads. Before additional filtering steps, various features of the insertion candidates, including the subfamily, target-site duplication, poly (A) tail, and TE structure, are annotated. Short-read sequencing data can be integrated before the local assembly step to perform a hybrid analysis [143].

Herein, Chu et al. created haplotype-resolve data using NA24385, one of the Ashkenazi Jewish trio samples characterized by the Genome in a Bottle (GIAB) [144]. They selected L1, *Alu*, and SVA insertions using RepeatMasker (https://www.repeatmasker.org/ (accessed on 3 August 2022)) and confirmed them as final TE insertions after checking the TSDs and poly (A) structures via the IGV browser [145]. In total, 197 L1, 1355 *Alu*, and 90 SVA novel insertions were identified. Afterward, they evaluated the sensitivity and specificity of the xTea with the benchmark data. In the case of the L1s, PacBio High-Fidelity (HiFi) showed 93%, PacBio continuous long reads (CLR) had 85%, and Nanopore achieved 87% sensitivity. However, Illumina showed 68% sensitivity. For specificity, PacBio HiFi, CLR, Nanopore, and Illumina showed 86%, 81%, 79%, and 93%, respectively. In general, PacBio HiFi had a relatively higher sensitivity than Illumina (91.3% vs. 80% on average), while Illumina had a higher specificity than PacBio HiFi (89% vs. 85% on average) [143].

The performance of the xTea in detecting germline TE insertions was evaluated compared to MELT. First of all, the high coverage (~300X) paired-end WGS data of NA24385 was realigned to the reference genome (hg38) and various sequencing depth ranges from 20X to 100X were used. The xTea showed higher F1 scores than MELT in L1 and *Alu* insertions in all coverages and a similar performance in SVA insertions. Second, the xTea was compared with the Transposon Finder in Cancer (TraFic-mem), used in analyzing somatic L1 insertions in cancer [23,146]. In 15 colon and paired-blood samples, the xTea identified 1671 somatic L1 insertions, including 277 transduction insertions, whereas TraFic-mem discovered 1103 L1 insertions with 200 transduction insertions. When used during a manual inspection of each candidate using an IGV browser, they have comparable rates (96% for the xTea and 97% for TraFic-mem) of insertion signature structures. Based on the haplotype-resolved data of NA24385, the xTea was compared with PALMER for long reads. Although PALMER showed an approximately 88% sensitivity in detecting *Alu* and SVA insertions, the xTea had an approximately 90% sensitivity in identifying L1, *Alu*, and SVA insertions. Moreover, the xTea outperformed PALMER by more than twice the specificity of detecting *Alu* and L1 insertions [143].

The long-read WGS data for 20 human individuals in the previous studies were further analyzed to detect full-length L1 near the centromere, HERV, pseudogene insertion, and TE-mediated SVs [147,148,149]. Despite the high quality of the human reference genome assembly, there are still hundreds of unknown regions, especially in the centromeres. Since epigenetic regulations in the centromere positively associate with the enriched repetitive satellite, centromere region could be a ‘land of plenty’ in full-length L1 retrotransposition [150]. Therefore, with the xTea using 20 long-read-sequenced genome data, they identified the full-length L1 of an average of nine groups per individual genome in the centromere region. In addition, it discovered 12 HERV insertion loci, 31 pseudogene insertions, and 78 SVs, including 48 deletions, 24 duplications, and 6 inversions. Indeed, the xTea might fail to notice some cases if there are insufficient clipped support reads, especially with low purity data. However, the xTea obtained higher accuracy in detecting germline or somatic TE insertions and various features that short-read-based tools could not detect. Taken together, the xTea has the potential to answer unsolved problems related to TE insertions in various genomic fields.

## 5. Concluding Remarks

Among the TEs that occupy almost 45% of the human genome, non-LTR retrotransposons, which move by a TPRT mechanism, have contributed to genetic diseases as some families still have retrotransposition activity. Furthermore, they are highly related to genetic disorders such as hemophilia A, leukemia, and breast cancer. With the growing need for insight into retrotransposon insertions, the advancement in NGS technologies leads the development of versatile bioinformatics tools capable of detecting retrotransposon insertions, including non-reference insertions, somatic insertions, complex TE-mediated SVs, and insertions in highly repetitive regions, especially the centromere region (Table 3). Given that recently developed long-read sequencing data tools can complement a variety of features that short-read-based tools have overlooked, future computational methods can be utilized to resolve a comprehensive understanding of human retrotransposons and their contribution in genomic/genetic changes. Furthermore, one day, those computational approaches focused on TE detection would be troubleshooters in closing the complete human or other genomes against the stubborn genomic regions.

## Figures and Tables

**Table 1:** Comparison of Illumina and MGI sequencing platforms.

Instrument		Run Time	Maximum Read Length	Maximum Reads	Output(Gb)	Key Applications	* Accuracy (>Q30)
Illumina	iSeq	9.5~19 h	2 × 150 bp	~4 million	1.2	microbe WGS, targeted gene sequencing	>80% of bases
	MiniSeq	4~24 h	2 × 150 bp	~25 million	~7.5	microbe WGS, targeted gene sequencing, targeted gene expression profiling	>80% of bases
	MiSeq	4~55 h	2 × 300 bp	~25 million	~15	microbe WGS, targeted gene sequencing, 16S metagenome sequencing	>75% of bases
	NextSeq 500	12~30 h	2 × 150 bp	~400 million	~120	microbe WGS, targeted gene sequencing, transcriptome sequencing	>75% of bases
	NovaSeq	~44 h	2 × 250 bp	~20 million	~6000	large WGS (human, animal, plant), single-cell profiling, transcriptome sequencing	≥75% of bases
MGI	MGISEQ-2000	12~78 h	2 × 200 bp	~1800 million	~1080	WGS, WES, targeted sequencing	≥75% of bases
	DNBSEQ-T7	24~30 h	2 × 150 bp	~5000 million	~6000	WGS, WES, transcriptome sequencing, targeted panel projects	>85% of bases
	DNBSEQ-G400	17~30 h	2 × 200 bp	~1800 million	~720	WGS, WES, transcriptome sequencing, microbial detection	>75% of bases
	DNBSEQ-G50	9~40 h	2 × 150 bp	~500 million	~150	microbe WGS, targeted DNA/RNA panels, forensic testing	>80% of bases

* Accuracy at maximum read length.

**Table 2:** Comparison of PacBio and nanopore sequencing platforms.

Instrument		Run Time	Read Length	Output	Application Features	Error Rate
PacBio	RS II	~4 h per SMRT cell	~15 kb	~10 Gb	WGS, targeted sequencing, metagenomics	13~15%
	Sequel	~20 h per SMRT cell				
	Sequel II	~30 h per SMRT cell		~500 Gb		
	Sequel IIe					
Oxford Nanopore	MinION	~72 h	>4 Mb	~50 Gb	WGS, WES, whole-transcriptome sequencing, metagenomics	5~13%
	GridION			~250 Gb	whole-transcriptome sequencing, metagenomics	
	PromethION			~14 Tb	population-scale genome sequencing, whole-transcriptome sequencing	

**Table 3:** Computational methods for detecting transposable element (TE) insertions from NGS data.

Name of Method	Detection Use and Target	Sequencing Type	Data Type	Sensitivity	Availability*/-(Accessed on 7 July 2022)	Ref
				(PCR-Based)		
RetroSeq	Non-reference TE insertions, genotype	WGS	Short read	>90%	https://github.com/tk2/RetroSeq	[119]
alu-detect	Non-reference *Alu* insertions	WGS, WES		>97%	http://compbio.cs.toronto.edu/alu-detect/	[123]
Tangram	Non-reference TE insertions, genotype	WGS		>94%	https://github.com/jiantao/Tangram	[125]
MELT	Population analysis of reference/non-reference TE insertions, genotype	WGS		>99%	http://melt.igs.umaryland.edu	[132]
iMGEins	Non-reference TE insertions in individual genomes	WGS		>96%	https://github.com/DMnBI/iMGEins	[133]
AluMine	Non-reference *Alu* insertions, missed *Alu* elements in reference, genotype	WGS	Raw short-read data	>98%	https://github.com/bioinfo-ut/AluMine	[135]
PALMER	Non-reference TE insertions, genotype	WGS	Long read	N/A	https://github.com/mills-lab/PALMER	[137]
xTea	Comprehensive analysis of non-reference and somatic TE insertions, genotype	WGS	Short or Long	>90%	https://github.com/parklab/xTea	[143]
			(Hybrid)			

## Data Availability

The data used to support this study are included in the manuscript.
